# Cardiac Myxoma as a Rare Cause of an Ischemic Stroke of the Vertebrobasilar Territory in a Young Adult: A Case Report

**DOI:** 10.7759/cureus.24792

**Published:** 2022-05-06

**Authors:** Sarra Saaf, Amal Miqdadi, Fatima-Zahra Merzouk, Karim El Aidaoui, Asmaa Hazim

**Affiliations:** 1 Neurology, Cheikh Khalifa International University Hospital, Mohamed VI University of Health Sciences, Casablanca, MAR; 2 Cardiology, Cheikh Khalifa University Hospital, Mohamed VI University of Health Sciences, Casablanca, MAR; 3 Anesthesia and Critical Care, Cheikh Khalifa International University Hospital, Mohamed VI University of Health Sciences, Casablanca, MAR; 4 Neurology, Cheikh Khalifa University Hospital, Mohamed VI University of Health Sciences, Faculty, Casablanca, MAR

**Keywords:** atrial fibrillation, cardiac myxoma, brainstem lesion, vertebrobasilar territory, young adult ischemic stroke

## Abstract

An ischemic stroke is characterized by the brutal installation of a focal functional deficit. Myxomas are the most common primitive cardiac tumors. Neurological manifestations of myxomas are mostly related to cardioembolic events, either caused by a migrating fragment of the tumor or by an attached clot that got detached from the tumor

This article describes the case of a 55-year-old male who presented with an ischemic stroke of the vertebrobasilar territory. Cardiac exploration revealed the presence of a cardiac mass. The patient was surgically treated and the anatomopathological study confirmed the diagnosis of cardiac myxoma. This case emphasizes the obligation to research the etiology of ischemic strokes, and more importantly the realization of a complete cardiologic exploration.

## Introduction

An ischemic stroke is characterized by the brutal installation of a focal functional deficit. Myxomas are the most common primitive cardiac tumors representing 80% of cardiac tumors [1]. They can manifest through multiple neurologic manifestations including ischemic stroke. Ischemic stroke is the most frequent neurologic manifestation of cardiac tumors [2]. They mostly result from a cardioembolic mechanism, resulting from a migrated blood clot or a migrated fragment from the tumor [3]. In the cases found in the literature, these ischemic strokes are mostly described in anterior circulation territories. This article aims to present the rare case of a 55-years-old man, who suffered from an ischemic stroke in the territory of the basilar artery six months after a prior ischemic stroke that happened on the same territory. This was found to be a consequence of an untreated cardiac myxoma during the following cardiac explorations. 

## Case presentation

A 55-year-old male patient, nonsmoker, was admitted to the emergency department due to sudden diminished strength of his left arm and leg associated with slurred speech 12 hours before his admission to the ED. The patient didn’t report any headache or convulsive crisis, nor loss of consciousness, with no particular context associated with the onset of the symptoms.

Previous medical history revealed arterial hypertension diagnosed six months ago, for which the patient is ongoing therapy with calcic inhibitors and angiotensin II inhibitors; an ischemic stroke of the vertebra-basilar territory six months ago with a minor motor deficit of his left upper limb; and a COVID-19 infection two weeks prior to his first ischemic stroke. The recovery was significant, with no deficit left.

Physical examination at the emergency department showed good general condition, blood pressure at 144/99 mmHg, heart rate at 69 beats/min, an oxygen saturation at 96 % and no fever. Neurologic examination revealed a conscious, oriented patient with left hemiparesis at 3/5, a mild paralytic dysarthria, facial central paralysis. Gag reflex was absent, and the patient reported an inability to drink water although he was able to eat solid food. Pulmonary and cardiac auscultation were normal.

**Figure 1:**
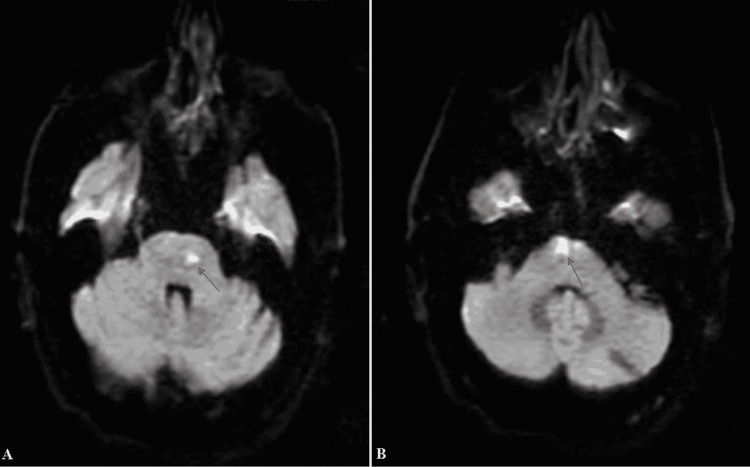
Axial diffusion brain MRI images Axial diffusion brain MRI shows an ischemic stroke in the pons (A) and in the anterior bulbar area (B)

**Figure 2:**
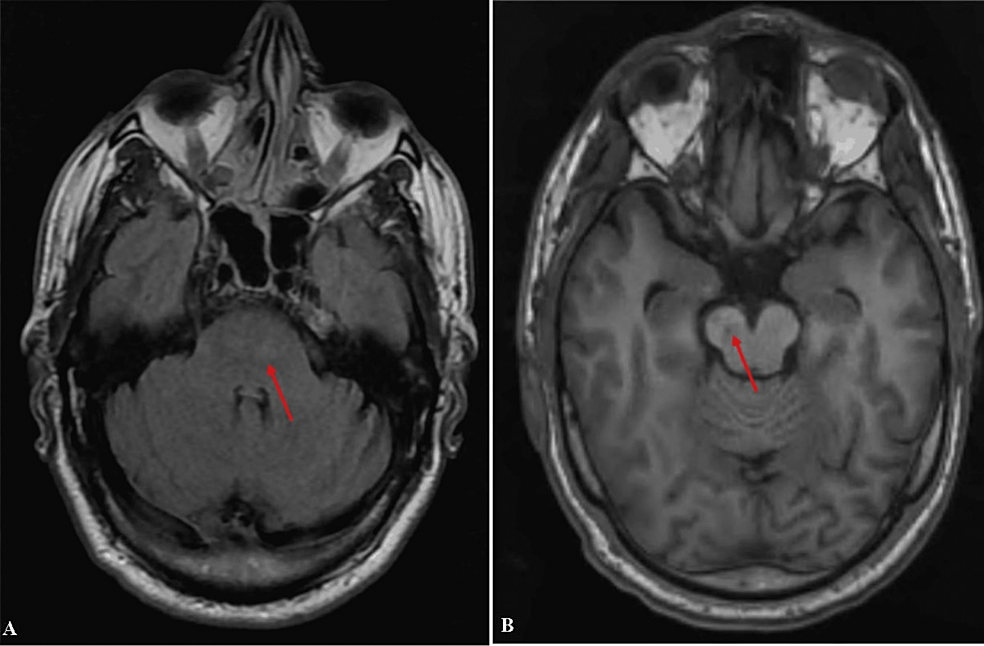
Axial Flair sequence cerebral MRI Cerebral MRI, Axial Flair sequence, showing a flair expression of the ischemic stroke in the pons (A) and the anterior cerebral peduncule (B)

**Figure 3:**
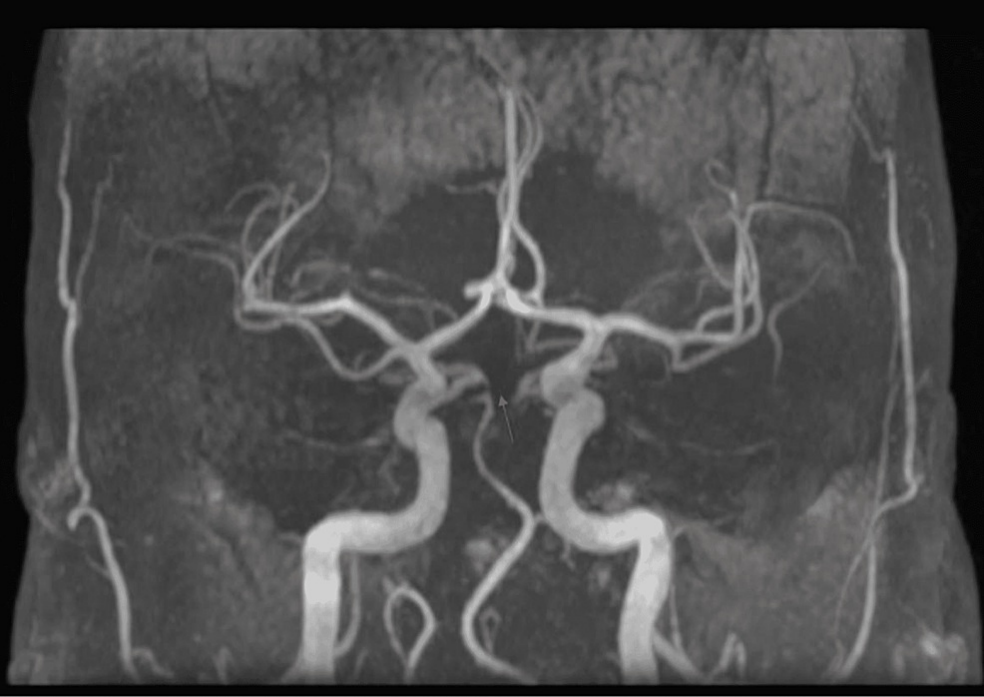
MRA image Magnetic resonance angiography (MRA) shows obstruction at the junction of the posterior cerebellar artery and the posterior cerebral artery

An ischemic stroke was highly suspected, and an MRI was performed as well as initial biologic tests. The MRI and magnetic resonance angiography (MRA) showed a left anterior bulbar ischemic stroke that extends to the left anterior pons (Figures 1-3). 

Initial blood work showed no particular alteration of hemogram, renal and hepatic function, and thyroid hormone. The electrophoresis of serum protein observed an inflammatory syndrome and the sedimentation rate was elevated. The patient was admitted to the cardiologic intensive care unit for surveillance as a safety measure because a cardioembolic etiology was highly suspected in regards of the localization of his ischemic lesion and clinical findings. In accordance to international regulations, antihypertensive therapy was stopped.

**Figure 4:**
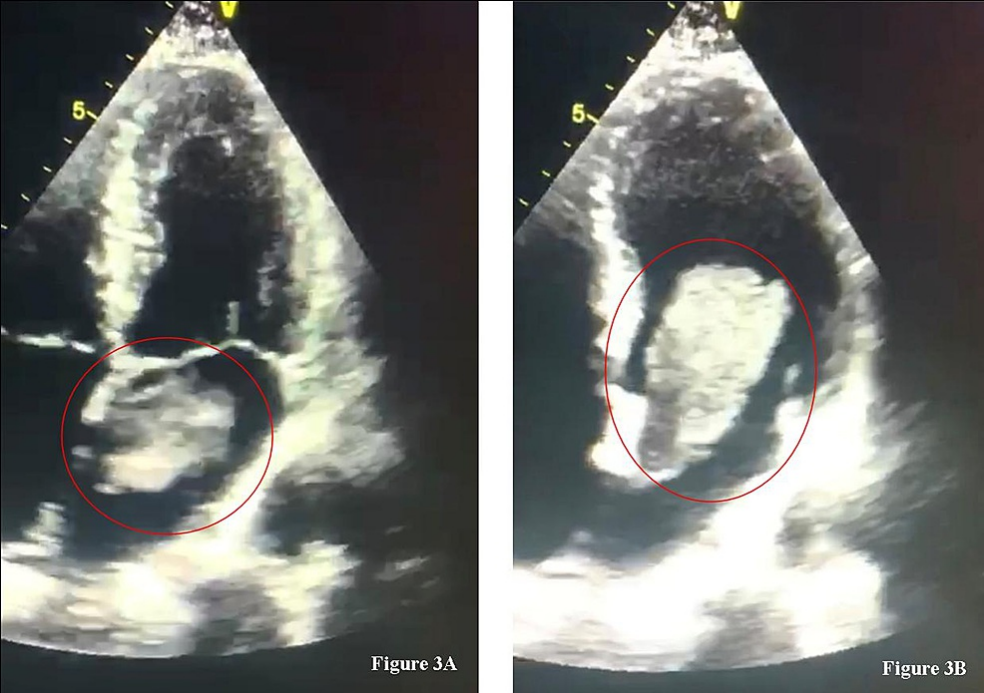
Echocardiographic findings Presence of a pedonculated mass in the left atrium (Figure 3 A) that penetrates the left ventricle during diastole (Figure 3 B)

The patient was tested for all the possible etiologies (thrombophilic, auto-immune and infectious) of ischemic strokes affecting young adults. A complete cardiac evaluation was performed. The transthoracic echocardiography observed a mobile mass (5,5 x 6 x 3 cm) located in the left atrium of the heart that penetrates the left ventricular chamber during diastole (Figure 4, Video 1). The mass does not appear to alter systolic function (ejection fraction of 71 %). 

**Video 1:** Echocardiographic view of a giant atrial myxoma

The thoracic scan showed no sign of secondary localization. The 24-hours electrocardiogram found no signs of cardiac rhythm abnormalities. An ophthalmologic exam was also performed as part of the etiological exams for young adult ischemic strokes, with no signs of vasculitis. 

The mass was suspected to be a myxoma although the certainty of the diagnosis can only be established after anatomopathological study. Cardioembolic origin is decided to be the most probable cause and anticoagulation therapy was started with an initial dosage of 4000 UI of enoxaparine a day associated with clopidogrel. A head-scan was performed 48 hours after the onset of anticoagulation therapy in search of a hemorrhage. The scan did not reveal an hemorrhagic complication

The patient was referred to the cardiothoracic surgery team based on the echocardiographic findings. A surgical indication was established, and the patient was prepared for surgery. Clopidogrel was stopped five days prior to the planned intervention (a week after the IS), and the patient was put under 4000 IU of enoxaparine once a day.

**Figure 5:**
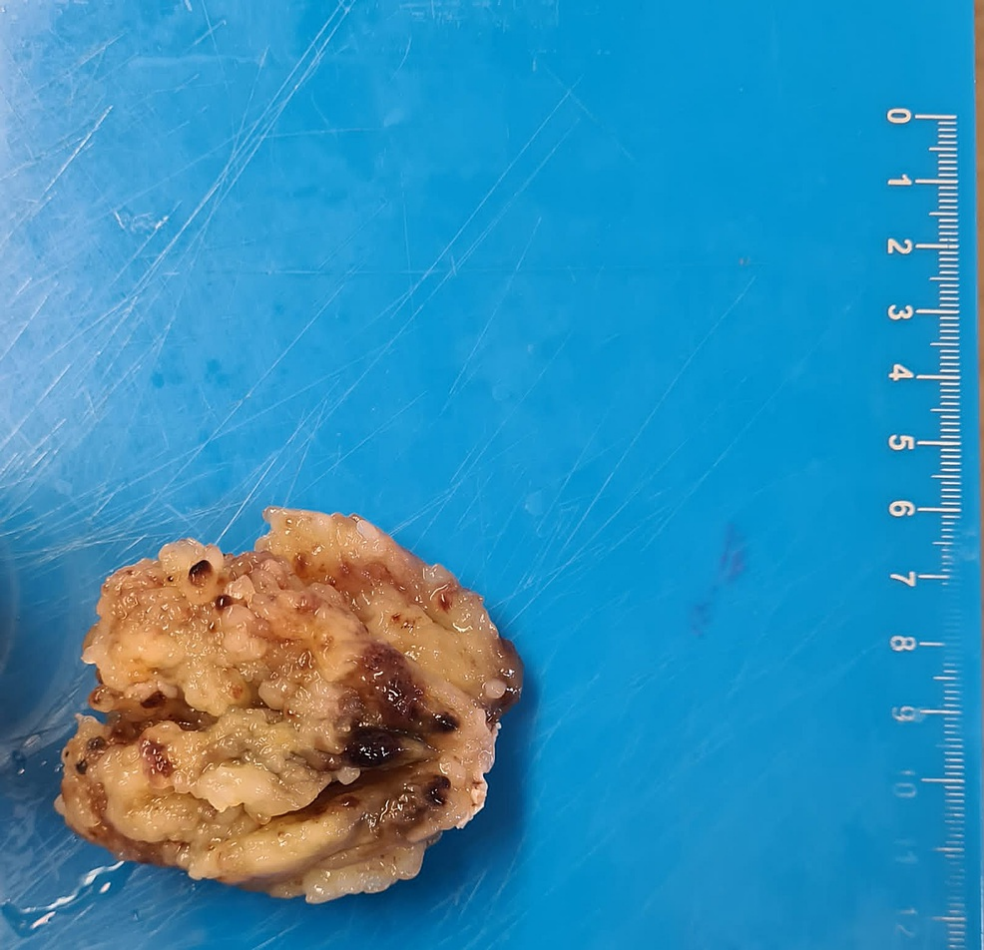
Macroscopic aspect of the resected mass

A head-scan was performed 48 hours after the onset of anticoagulation therapy to detect a brain hemorrhage. No hemorrhage was found. The surgical procedure was successful and the myxoma was fully resected (Figure 5), with no neurological complications at the patient’s awakening from anesthesia in the days following surgery. 

However, twenty-four hours after surgery, the patient developed an atrial fibrillation that lasted for more than 24-four hours on the EKG monitoring during the patient’s stay in cardiologic intensive care unit. The anatomopathological examination of the tumor confirmed the myxoma’s diagnosis with the presence of typical cells (stellar and fusiform cells with a central nucleus containing fine chromatin and a subtly nucleolated and a clear cytoplasma).

## Discussion

The incidence of ischemic stroke in young adults is rising since the 1980s [4]. This category of ischemic strokes distinguishes itself by the multiple etiologies that they may result from which are not common in older patients. The identified cause of ischemic stroke that has been most frequently encountered in young adults is arterial dissection [5], although in most cases it is cryptogenic with no identified etiology [6]. Ischemic strokes result from cardioembolic events in approximately 20 to 30% of the time [7]. Those are mostly related to cardiac rhythm abnormalities which lead to a blood clot migrating through blood vessels and occluding them. Cardioembolic events are likely to repeat themselves during the months following the first episode [8]. 

Myxomas are the most frequent benign cardiac tumor (80%). Seventy-five percent of all cardiac myxomas are found in women. They are mostly diagnosed in adults in their forties and fifties with a median age of 56 years [1]. Although mostly asymptomatic, their clinical manifestations depend on their size and the potential repercussions they might have on valvular function, distant arterial embolisms, or other nonspecific symptoms [9]. They present with neurological manifestations in up to 25 % of the cases, predominantly in the form of an ischemic stroke or a transient ischemic attack, but other manifestations can be found in the form of a cerebral hemorrhage [2].

Neurological manifestations of myxomas are mostly related to cardioembolic events, either caused by a migrating fragment of the tumor or by an attached clot that got detached from the tumor [3,10]. The territory affected by the ischemic stroke adds relevance to this case. The patient had a brainstem ischemic stroke. The brainstem is supplied by the posterior circulation arteries. Basilar artery ischemic strokes represent 20-25% of ischemic strokes and cardioembolic events are responsible for 40% of these strokes [11]. Brainstem lesions that are secondary to cardiac myxomas account for 4.8 % of the cases reported [12], and the posterior circulation arteries are involved in 5% of the cases [13]. The patient presented with an ischemic stroke of the pons and the brainstem, supplied by the basilar artery which revealed his underlying myxoma after investigation. The patient’s history found a previous ischemic stroke episode in the same site, left unattended, which is in favor of a cardio-embolic event. 

The patient was tested for all possible etiologies that can be related to a young adult ischemic stroke, mainly for auto-immune and thrombophilic diseases as well as infectious diseases. These all came back negative, with the exception of the biological inflammatory syndrome found with his elevated sedimentation rate and the electrophoresis of blood proteins which was in favor of an unspecified inflammatory syndrome. The presence of this inflammatory syndrome supports the diagnosis of cardiac myxoma, as it is the only cardiac tumor that is associated with systemic manifestations, probably due to the fact that myxomas exude interleukin (IL)-6 [14]. 

Surgical resection of myxomas must be performed promptly to prevent the complications associated with myxoma, mainly systemic embolization [1]. In this case, it was necessary because of the dual risk of a new neurologic manifestation and an obstruction of the mitral valve due to the tumor’s size. 

Atrial fibrillation is the most common cardiac rhythm disturbance that can occur following heart surgery. It usually appears in the first two days following the procedure [15]. Our patient presented with atrial fibrillation for 24 continuous hours. The anticoagulation treatment given to the patient at his departure from the hospital was adapted to this development. 

## Conclusions

Primary cardiac tumors are an extremely rare cause of cardioembolic event that would cause a posterior cerebral territory ischemic stroke. The neurological and cardiac prognosis are good. This case emphasis the obligation to research the etiology of ischemic strokes, and more importantly the realization of a complete cardiologic exploration.
